# Determining appropriateness for rehabilitation or other subacute care: is there a role for utilisation review?

**DOI:** 10.1186/1743-8462-4-3

**Published:** 2007-03-13

**Authors:** Christopher J Poulos, Kathy Eagar

**Affiliations:** 1 South Eastern Sydney and Illawarra Area Health Service, PO Box 21 Warrawong, NSW, 2502, Australia; 2 Centre for Health Service Development, University of Wollongong, NSW, 2515, Australia

## Abstract

**Background:**

Rehabilitation and other forms of subacute care play an important role in the Australian health care system, yet there is ambiguity around clinical definitions of subacute care, how it differs from acute care, where it is best done and what resources are required. This leads to inconsistent and often poorly defined patient selection criteria as well as a lack of research into efficient models of care.

**Methods:**

A literature review on the potential role of utilisation review in defining levels of care and in facilitating appropriate care, with a focus on the interface between acute care and rehabilitation.

**Results:**

In studies using standardised utilisation review tools there is consistent reporting of high levels of 'inappropriate' bed days in acute care settings. These inappropriate bed days include both inappropriate admissions to acute care and inappropriate continuing days of stay. While predominantly an instrument of payers in the United States, concurrent utilisation review programs have also been used outside of the US, where they help in the facilitation of appropriate care. Some utilisation review tools also have specific criteria for determining patient appropriateness for rehabilitation and other subacute care.

**Conclusion:**

The high levels of 'inappropriate' care demonstrated repeatedly in international studies using formal programs of utilisation review should not be ignored in Australia. Utilisation review tools, while predominantly developed in the US, may complement other Australian patient flow initiatives to improve efficiency while maintaining patient safety. They could also play a role in the identification of patients who may benefit from transfer from acute care to another type of care and thus be an adjunct to physician assessment. Testing of the available utilisation review tools in the Australian context is now required.

## Background

### Introduction

Rehabilitation and other subacute care plays a significant role in the Australian health care system, providing a valuable contribution to patient outcomes and being essential for the flow of patients from acute care. Yet there is ambiguity around what subacute care is, how it differs from acute care or other longer term care such as 'transition care', where it is best done and what resources are required. While rehabilitation is perhaps the most readily recognised type of subacute care, with well-accepted service models, there still remains inconsistency when it comes to patient selection for rehabilitation.

This paper briefly considers the concept of subacute care, but with a particular emphasis on patient selection for rehabilitation and on the interface between acute care and rehabilitation. It then examines the role that formal utilisation review may have in an acute hospital in the identification of patients who may be more appropriately classified as requiring a subacute level of care, including rehabilitation. If utilisation review can be shown to offer assistance in clinically defining the boundary between acute and subacute care, then research into models of subacute care, including optimising the interface between acute and subacute care, may be facilitated. Utilisation review could also provide a mechanism for improving the transit of patients within acute care, by assisting in the identification of inefficiencies in the processes of care. These issues are relevant to clinicians, hospital administrators and policy makers.

### Subacute care and rehabilitation

Eagar and Innes introduced the term 'subacute' into Australia in 1992 to describe patients whose need for health care is predicted by their functional status, rather than their principal medical diagnosis [1]. Other definitions of subacute care also exist. Common to all is that there is a group of patients who no longer meet criteria for classification as 'acute', but who still require care in a hospital setting, with the care required being more clinically intense and goal directed than is long term care [2-5]. The issue becomes more difficult when trying to define the actual boundary between acute care and subacute care, with the situation in Australia being one where, according to Eagar and Innes, our 'acute' hospitals "treat a diverse population of patients, many of whom would not meet criteria for classification as acute" [1]. In a later paper, Eagar then discusses the boundaries between acute care and other forms of care, and the development of the subacute and non-acute patient casemix classification system [6].

In Australia, rehabilitation is classified, for casemix purposes, as a distinct form of subacute care [7]. The AN-SNAP (Australian National Sub-acute and Non-Acute Patient) classification system, developed in 1997, defined four types of subacute care (Rehabilitation, Geriatric Evaluation and Management, Psychogeriatric and Palliative Care), as well as non-acute (Maintenance) care, with these definitions being subsequently incorporated into the National Minimum Data Set for Admitted Patient Care [8]. Within AN-SNAP, a rehabilitation episode of care is one that is: 'provided for a person with an impairment, disability or handicap' and; 'for whom the primary treatment goal is improvement in functional status' and; 'which is evidenced by an individualised and documented initial and periodic assessment of functional ability by the use of a recognised functional assessment measure' and 'an individualised multidisciplinary rehabilitation plan which includes negotiated rehabilitation goals and indicative time frames' [9].

While the current Australian definitions that exist for subacute care, including rehabilitation, may be useful for casemix purposes and to describe the general characteristics of this patient population, they are not as helpful when trying to prospectively identify patients who may be appropriate for such care or for determining when it should commence. This, in turn, leads to an inability to examine different models of care for such patients. Eagar (1999) notes that the boundary between acute care and rehabilitation needs to be more clearly defined now that there is a classification system for rehabilitation and subacute care [7].

A 2001 Victorian Department of Human Services report into the interface between subacute and acute care [10] noted that there was a 'lack of focus and coordination in referral to, and provision of, subacute services, which affects throughput and efficiency'. The report raised the issue of the timing of patient transfer between acute and subacute services, and the impact that may have on both the acute and subacute episode. While the report details strategies to address some of these issues, the use of more transparent and validated patient selection criteria for rehabilitation and other subacute care was not mentioned.

### The interface between acute care and rehabilitation

Rehabilitation medicine services within Australia generally have guidelines, either implicit or explicit, that broadly define the types of patients that they will accept for an inpatient rehabilitation program. These guidelines will usually include clinical factors, such as the potential for the patient to functionally improve with rehabilitative therapy, the capacity of the patient to participate in a rehabilitation program and the degree to which the patient is medically stable. Other factors may include specific goals of the patient and/or carers and the patient's premorbid level of functioning.

In practice, while the decision about if, and when, to transfer a patient to a rehabilitation bed is largely based on the clinical judgement of the assessing rehabilitation physician or registrar, the threshold for accepting a patient for rehabilitation is often influenced by a number of system factors. These may include the degree of 'bed pressure' in acute care, the availability of the rehabilitation bed, access to diagnostic investigations and/or ongoing medical or surgical care or review in the rehabilitation facility, and the availability of substitutable ambulatory rehabilitation programs.

Transferring patients from acute care to rehabilitation or other subacute level of care at the optimal time may have significant benefits, both for the patient, as well as for the health system [10]. Outcomes for patients may be improved if they are able to commence formal rehabilitation earlier and there may also be improvements in overall hospital length of stay and cost of care. In addition, the problem of 'access block' may be helped by the more timely transfer of patients from acute care beds to rehabilitation. Conversely, there may be adverse outcomes if patients are transferred too early. For example, patients who remain medically unstable may not be able to be safely managed in the rehabilitation facility, unstable medically conditions could render the rehabilitation process less effective, and undue time could be wasted if the patient has to be transferred back to the acute care facility, or other centre, for diagnostic or medical evaluation.

Selection for a formal rehabilitation program is relatively clear-cut when patients have the new onset of defined impairments that are likely to be responsive to rehabilitation. The situation is less clear when patients have multiple morbidities or general debility and this group, which is typically older patients, is increasingly occupying acute care wards as the population ages. These patients will often have completed an acute episode, are no longer deemed to require acute care by their medical or surgical teams, but are not able to be discharged. They often require a period of restorative care and/or complex discharging planning, with the question often becoming whether transfer to a formal rehabilitation or subacute/post-acute program is the best option, or whether the patient is more efficiently managed by remaining in the acute care ward until ready for discharge.

One way of more clearly defining the boundary between acute care and rehabilitation or other subacute care is to develop specific criteria for the identification of patients who no longer meet criteria for classification as 'acute', as well as selection criteria and processes for rehabilitation transfer. This suggests a role for utilisation review.

## Methods

A Medline search was conducted via Ovid to examine the literature on selection for rehabilitation or other subacute care and the role of utilisation review in these situations. Key words searched included utilization review, rehabilitation, physical medicine, subacute care, and patient selection. These returns were screened by title, initially for relevance to "rehabilitation" or "sub-acute care" and "utilization review" or "patient selection". Those not evident from the title were reviewed at abstract level for relevance. The references from each of the chosen papers were then reviewed to find other contributory papers. A general Internet search was also conducted, in addition to use of unpublished data from the Australian Rehabilitation Outcomes Centre (AROC), University of Wollongong, Australia.

## Results

### Selection Criteria for Rehabilitation

There is a growing literature on the predictors of rehabilitation outcomes, but selection criteria based only on those for whom a 'good' outcome can be anticipated will deny many patients the opportunity to achieve worthwhile functional recovery. Wade (2003) notes that purchasers of health care services often ask service providers to produce selection criteria. These are meant to ensure that only patients likely to benefit from an intervention are referred and accepted, and that all applicable patients are referred. However, in the rehabilitation context, Wade argues that the question of potential benefit is not always clear cut, with the situation being more a case of patients varying along two continua – the likelihood of benefit and the extent of benefit. He cautions against the use of public selection criteria, due to the lack of good evidence on who is responsive to rehabilitation and the danger of asking untrained staff to apply clinical criteria [11].

Much of the literature on patient factors that predict a good rehabilitation outcome centres on specific diagnostic groups, such as stroke or orthopaedic conditions [12,13], but selection for rehabilitation becomes less clear when patients have multiple morbidities or general debility [14]. This would seem to be an increasing trend in Australia, as unpublished AROC data show that up to 25% of rehabilitation episodes in public hospitals are now for patients with more general debility or multiple impairments. But there is also anecdotal evidence to suggest that, even with relatively straightforward conditions such as elective joint replacement, the utilisation of formal rehabilitation programs varies widely between the states and between the public and private sectors. If this is the case, a lack of uniform patient selection criteria may be a factor.

There is very little in the literature on formal criteria or procedures for patient selection for rehabilitation and little evidence to guide the development of such criteria. This deficit has been recognised, with Unsworth (2001) [12] noting that objective criteria for the selection of patients for rehabilitation may help acute care clinicians make more informed discharge planning decisions.

Alternatives to physician assessment alone for selection for rehabilitation have been explored. For example, members of the multidisciplinary rehabilitation team could be involved in the selection process. One US study showed that rehabilitation outcomes for stroke patients were the same if patients were selected by a physiatrist (rehabilitation physician) alone, or by the physiatrist basing their decision to accept a patient on a nurse practitioner's assessment [15]. However, the reliability of the clinical judgement of different members of the multidisciplinary rehabilitation team in determining the rehabilitation potential of patients has been questioned, with the suggestion that, in the case of older patients, it may be preferable to use a standardised assessment in the initial decision regarding patient selection [14]. Other suggestions include scoring systems to determine the site of rehabilitation (home versus post-acute facility) for patients following total hip replacement [16], or nurse to nurse referral for rehabilitation in community hospitals in the United Kingdom [17].

The issue of selection criteria for other 'subacute' care is less clear than for rehabilitation, probably because definitions of what constitutes subacute care vary [18].

### Utilisation Review – a brief description

Utilisation review is a method that assesses the appropriateness of the medical or clinical care provided to a patient, including the appropriateness of the care setting and the duration of care [19]. Inappropriate hospital utilisation includes both over- and under-utilisation. Over-utilisation includes the admission to hospital of patients who could have been managed, from a clinical perspective, in a less intensive care setting, or patients who remain in a more acute setting for longer than required [20]. Under-utilisation occurs when patients do not receive the intensity of care required.

Utilisation review information is derived from the patient's medical record, their treating clinical team, or a combination of these sources. Concurrent utilisation review is the most common, as well as the most useful, as it allows for corrective action to be taken, such as discharge planning or finding a more appropriate care setting for the patient. Retrospective reviews are likely to reveal higher rates of inappropriate utilisation than concurrent reviews, but this is usually due to information justifying a level of care being missing or unavailable [21].

The utilisation review literature consistently demonstrates high levels of inappropriate hospital bed days for patients in acute care, with a large percentage of these days being for patients who should, according to the review criteria, be in a lower level of care. The reported rate of inappropriate days of stay in acute care ranges from around 19% to 60%, while between 18% and 48% of admissions to acute care have been reported as inappropriate [5]. Causes of inappropriate days of stay include delays in the discharge process, lack of appropriate post acute care services, delays in diagnostic tests, and delays in medical and other specialised consultations [22]. Utilisation review tools may also highlight situations when the patient remains in acute care when the need is for rehabilitation or other subacute level of care.

There can also be significant rates of under-utilisation of acute care, although there are fewer studies available that specifically examine under-utilisation. The amount of inappropriate under-utilisation is reported as being much smaller (less than 4 %) than that for inappropriate over-utilisation [23]. Detecting under-utilisation may assist in maintaining clinical quality by the monitoring of premature discharge, or care in a sub-optimal setting (for example, when patients should be in critical care rather than on a general ward, or the premature transfer to rehabilitation of patients who are medically unstable).

Utilisation review became widespread in the United States following the introduction of Medicare and Medicaid [19]. Utilisation review programs have since been adopted in Canada, the United Kingdom, and Europe, but less so in Australia [24,25]. In the US, formal utilisation review programs have primarily become a tool of payers of health care services to better manage costs. However, another cited reason for detecting over-utilisation is to help reduce the iatrogenic risk associated with hospitalisation [19]. Done concurrently, utilisation review in the United States is regarded by managed care organisations as being both a cost containment strategy and a quality improvement tool [26]. However, outside of the United States, utilisation review tools are seen more as an aid to facilitate appropriate care, rather than a mechanism for approving or denying care, or the payment for care, for individual patients [27].

When utilisation review was introduced, appropriateness was based primarily on the reviewer's judgement. However, when inter-rater reliability was found to be inadequate, even when using physicians who had been selected based on their expertise, attention was placed on the development of specific criteria. The Appropriateness Evaluation Protocol (AEP) by Gertman and Restuccia [28] was the most widely used tool initially developed. The AEP contained a list of medical and nursing/life services that were judged to be only available at an acute hospital and a list of patient condition factors that were thought to require the resources of an acute hospital. A patient day was considered appropriate if any one of the services or conditions was present [19].

While utilisation review may be able to detect 'inappropriate' days of stay in acute care, it remains only an assumption that patients will be more appropriately managed in a less acute setting [29]. Further, there is evidence that only about 50% of unnecessary days in acute care can be avoided without additional resources being required, and that the 'inappropriate' days are less resource intensive and thus less costly [30]. This needs to be considered when determining the cost effectiveness and clinical appropriateness of utilisation review interventions. In addition, because overall hospital length of stay in acute care has fallen, it is possible that there may not be as many inappropriate days of stay now, compared to the findings of earlier studies.

Utilisation review has not been widely reported as a tool to assist in the determination of the appropriateness for, and timing of, transfer to rehabilitation or subacute care. While a number of utilisation review tools are reported in the literature, very few tools report specific criteria for determining appropriateness for rehabilitation and subacute care. The three tools reported to include selection criteria for rehabilitation or sub-acute care are all proprietary products. These are the InterQual Criteria (McKesson Corporation), the Managed Care Appropriateness Protocol – MCAP – which is based on the AEP (The Oak Group), and the Milliman Care Guidelines (Milliman USA). Being proprietary, access is not freely available, and there is only limited information available on them in the peer reviewed literature. Of these three, the InterQual Criteria is the most widely reported, with about 25 papers or citations in Medline.

### The InterQual Criteria – a utilisation review tool

The InterQual Criteria is a proprietary utilisation review tool developed in the United States. It has been cited in published work originating from both the US and outside the US [5,23,24,27,29,31-46]. For example, one US study (a retrospective chart review of 858 admissions) used the InterQual subacute criteria to determine the prevalence of subacute patients in acute care beds in 43 Veterans Affairs Hospitals in the US. This study showed that over one third of patients (38%) had at least one subacute day during their acute admission, with subacute days occurring more frequently for medical (42%) than for surgical admissions (33%). For those admissions which had any subacute days, 54% of the days in acute care were classified as subacute by the InterQual Criteria [31]. This was equivalent to almost 7 bed days per admission. This study also found that patients experiencing subacute days were likely to be older and sicker. The authors suggest that future studies focus on developing targeting criteria that enable clinicians to prospectively identify patients with subacute care needs. The authors also note that the purpose of subacute care is not just to move patients from one setting to another, but to provide more appropriate care.

Published papers outside of the United States indicate that the InterQual Criteria have been predominantly used in Canada and the United Kingdom [5,23,29,32,33,39,40,42,46,47]. DeCoster et al (1997), in a retrospective chart review of 3,904 patients in Canada, found that, after one week, 53.2% of patients assessed as needing acute care on admission no longer required acute care. Patients 75 years of age accounted for more than 50% of bed days, but 74.8% of these bed days were regarded as being inappropriate for acute care. The authors noted that the InterQual Criteria have the advantage of being diagnosis independent (thus being unaffected by diagnostic errors), they are broadly accepted by physicians as being a reasonable measure of the need for acute care, and they have been externally validated[33].

In another large Canadian study involving 189 acute care hospitals and 13,242 patient discharges, Flintoff et al (1998) used the InterQual (Adult Acute) criteria to determine the level of care most appropriate for admission and subsequent days of stay [5]. They found that, for all admissions, 62.2% were judged by the criteria to be acute, 19.7% subacute and 18.1% non-acute. Following admission, acute care was needed on only 27.5% of subsequent days, subacute care on 40.2% of days and non-acute care on 32.3% of days. Inter-rater reliability in this study was found to be high (kappa ranged from 0.71 to 1.00).

When used in the United Kingdom, the InterQual Criteria were found to have high reliability and to be valid when there was a presumption that the full range of alternative levels of care was available. There were limits to their validity in the UK National Health Service when the alternatives were not available [40], leading to the criticism that, if the alternatives are not available, then utilisation review is not achieving its aims [48]. However, it is also suggested that health services planners could use the information supplied by the utilisation review process to then evaluate the benefits of developing those services which are not available [47].

Utilisation review, and the various review tools, are not without their critics, with concerns raised about the validity of the criteria being used [29,36,37,49,50]. The InterQual tool, along with the AEP, was shown to have moderate validity and reliability in the United States in a study done by Straumwasser in 1990, leading the authors to conclude that payment should not be denied based on the instrument alone, but only if the decision is confirmed by a physician [41]. Even though criteria such as InterQual have been validated against expert panels, the question arises as to how valid they remain with subsequent revisions and with changes in clinical practice. Also, validity may vary between institutions, depending upon the services available [34]. It should be noted, however, that administered concurrently, the InterQual Criteria allow for physician over-ride to the outcome of the review if there are clinical reasons for doing so.

### Applicability of utilisation review tools in Australia

While the concept of utilisation review is likely to be as applicable in Australia as it is in other developed countries, the applicability of the specific tools requires formal testing. The AEP has been trialled in an Australian study that audited admission appropriateness to an acute hospital, finding that it was both efficient and clinically valid for use in Australian hospitals, with only minor modifications required [25]. A further study, also using the AEP, found that 15.2% of admission days and 28.7% of days of stay were non-acute. The authors concluded that the AEP was a useful tool for assessing non-acute days of stay, but that inpatient treatment in acute care facilities in Australia may not be as rigidly controlled as in the US, where the tool was developed [51]. Despite these studies, adoption of the AEP in Australia as a utilisation review tool does not appear to have occurred.

One of the criticisms of the InterQual Criteria is its reduced suitability outside of the United States due to the existence of fewer alternatives to acute care available in other health systems [24]. Also, what constitutes 'acute care' may also differ, with the US appearing to have tighter definitions than in Australia as to what comprises acute care, with these definitions both shaping, as well as being shaped by, utilisation review tools.

## Conclusion

Tools to inform patient selection decisions, and which help to validate care within settings, are of relevance to clinicians, administrators and policy makers. While subacute care is an accepted and important component of the Australian health care system, it remains poorly defined from a clinical perspective. This lack of clinical definition impedes research into models of subacute care, including how it should best interface with acute care and when and how it should occur outside of the acute care setting.

Rehabilitation is a type of subacute care with firmly established models of clinical practice and good evidence of efficacy in a range of impairments. Yet patient selection for rehabilitation remains variable, relying predominantly on clinical judgement and being influenced by system factors such as rehabilitation bed availability and pressure on acute care. It is the challenge of our health care system to ensure that the potential gains to be made from multidisciplinary, goal directed, rehabilitation interventions are afforded to all patients likely to benefit.

This leads to a possible role for utilisation review. The high levels of 'inappropriate' care demonstrated repeatedly in international studies using a variety of tools, as well as the limited Australian work available, should not be ignored in Australia, especially as we grapple with issues of efficiency and patient safety. Yet formal utilisation review has not been embraced. Practiced overseas, utilisation review has a role in clinically determining the most appropriate level of care for an individual patient, with some tools also having specific criteria for selection for rehabilitation or other subacute level of care. As well as being potentially useful at the interface between acute care and other types of care, utilisation review has the potential to provide a mechanism by which the processes of acute care could be improved. It could also assist health service planners in determining acute and subacute capacity.

In the absence of well-validated, contemporary, public domain tools, there appears little choice but to consider proprietary utilisation review tools. The companies promoting them claim that the tools enhance efficiency and patient safety through having evidence-based checklists that support the safe transit of patients through different levels of care and care settings. However, the tools also have their critics and need to be tested against current Australian practice. Their applicability in the Australian context, where there are less alternate care settings than are available in the US, and where clinical terminology differs from the US, also needs to be tested.

Even if the tools are shown to be applicable in Australia, it would still need to be shown whether the establishment of formal utilisation review programs is cost effective, and whether these US-based systems are transferable to Australia without major modifications to the criteria and supporting software. The degree of physician acceptance is another very important issue. These are important research questions that need to be tested and which could have significant health policy implications for Australia.

## Competing interests

The author(s) declare that they have no competing interests.

## Authors' contributions

CP undertook the literature review. Both authors drafted the manuscript and approved the final manuscript.
